# Attenuation of Reactive Gliosis Does Not Affect Infarct Volume in Neonatal Hypoxic-Ischemic Brain Injury in Mice

**DOI:** 10.1371/journal.pone.0010397

**Published:** 2010-04-28

**Authors:** Katarina Järlestedt, Catherine I. Rousset, Maryam Faiz, Ulrika Wilhelmsson, Anders Ståhlberg, Hana Sourkova, Marcela Pekna, Carina Mallard, Henrik Hagberg, Milos Pekny

**Affiliations:** 1 Perinatal Center, Institute of Neuroscience and Physiology, Sahlgrenska Academy, University of Gothenburg, Gothenburg, Sweden; 2 Institute of Reproductive and Developmental Biology, Imperial College, London, United Kingdom; 3 Department of Obstetrics and Gynecology, Sahlgrenska Academy, University of Gothenburg, Gothenburg, Sweden; 4 Department of Medical Chemistry and Cell Biology, Institute of Biomedicine, Sahlgrenska Academy, University of Gothenburg, Gothenburg, Sweden; 5 Center for Brain Repair and Rehabilitation, Department of Clinical Neuroscience and Rehabilitation, Institute of Neuroscience and Physiology, Sahlgrenska Academy, University of Gothenburg, Gothenburg, Sweden; Brigham and Women's Hospital, Harvard Medical School, United States of America

## Abstract

**Background:**

Astroglial cells are activated following injury and up-regulate the expression of the intermediate filament proteins glial fibrillary acidic protein (GFAP) and vimentin. Adult mice lacking the intermediate filament proteins GFAP and vimentin (*GFAP^−/−^Vim^−/−^*) show attenuated reactive gliosis, reduced glial scar formation and improved regeneration of neuronal synapses after neurotrauma. *GFAP^−/−^Vim^−/−^* mice exhibit larger brain infarcts after middle cerebral artery occlusion suggesting protective role of reactive gliosis after adult focal brain ischemia. However, the role of astrocyte activation and reactive gliosis in the injured developing brain is unknown.

**Methodology/Principal Findings:**

We subjected *GFAP^−/−^Vim^−/−^* and wild-type mice to unilateral hypoxia-ischemia (HI) at postnatal day 9 (P9). Bromodeoxyuridine (BrdU; 25 mg/kg) was injected intraperitoneally twice daily from P9 to P12. On P12 and P31, the animals were perfused intracardially. Immunohistochemistry with MAP-2, BrdU, NeuN, and S100 antibodies was performed on coronal sections. We found no difference in the hemisphere or infarct volume between *GFAP^−/−^Vim^−/−^* and wild-type mice at P12 and P31, i.e. 3 and 22 days after HI. At P31, the number of NeuN^+^ neurons in the ischemic and contralateral hemisphere was comparable between *GFAP^−/−^Vim^−/−^* and wild-type mice. In wild-type mice, the number of S100^+^ astrocytes was lower in the ipsilateral compared to contralateral hemisphere (65.0±50.1 *vs*. 85.6±34.0, p<0.05). In the *GFAP^−/−^Vim^−/−^* mice, the number of S100^+^ astrocytes did not differ between the ischemic and contralateral hemisphere at P31. At P31, *GFAP^−/−^Vim^−/−^* mice showed an increase in NeuN^+^BrdU^+^ (surviving newly born) neurons in the ischemic cortex compared to wild-type mice (6.7±7.7; n = 29 versus 2.9±3.6; n = 28, respectively, p<0.05), but a comparable number of S100^+^BrdU^+^ (surviving newly born) astrocytes.

**Conclusions/Significance:**

Our results suggest that attenuation of reactive gliosis in the developing brain does not affect the hemisphere or infarct volume after HI, but increases the number of surviving newborn neurons.

## Introduction

The central nervous system (CNS) contains abundance of astroglial cells which induce formation of neuronal synapses and support neurons structurally and metabolically [1]. Astrocytes become activated by many pathological conditions such as neurotrauma, stroke, perinatal asphyxia or neurodegenerative diseases. One of the hallmarks of astrocyte activation and the resulting reactive gliosis is the upregulation of the intermediate filament (IF) system (known also as nanofilament system) composed of glial fibrillary acidic protein (GFAP), vimentin, nestin and synemin [2], [3]. The function of reactive astrocytes in neuroprotection or recovery of the CNS from an injury is not fully understood. Neuroprotection by reactive astrocytes through up-regulation of glutathione was demonstrated following oxidative stress [4], [5]. On the other hand, reactive gliosis may inhibit neuroregeneration and outgrowth of axons and dendrites [6].

Mice lacking the IF proteins GFAP and vimentin (*GFAP^−/−^Vim^−/−^*) showed that up-regulation of astrocyte IF is a key step in activation of astrocytes and that reactive gliosis is important for the wound healing process [3], [7], [8], [9]. Reactive astrocytes in *GFAP^−/−^Vim^−/−^* mice are unable to form any cytoplasmic IFs since neither nestin nor synemin can self-polymerize or co-polymerize in the absence of both GFAP, and vimentin [2], [10]. After neurotrauma, *GFAP^−/−^Vim^−/−^* mice show attenuated reactive gliosis with reduced hypertrophy of astrocyte processes and increased synaptic loss in the acute stage of the injury [8], [11], however, the regeneration of neuronal synapses at a later stage is improved [11]. Focal brain ischemia induced by middle cerebral artery transection led to increased infarction in *GFAP^−/−^Vim^−/−^* mice, which suggested protective role of reactive astrocytes in adult brain ischemia.

Here we subjected *GFAP^−/−^Vim^−/−^* mice to unilateral hypoxia-ischemia at postnatal day 9 [12], [13], [14] to address the importance of astrocyte IFs and reactive gliosis in perinatal asphyxia. We found no difference in the hemisphere or infarct volume between *GFAP^−/−^Vim^−/−^* and wild-type mice. However, the *GFAP^−/−^Vim^−/−^* mice showed a larger number of surviving newly born neurons. In contrast to wild-type mice, we did not find a loss of S100^+^ astrocytes in the ischemic hemisphere of *GFAP^−/−^Vim^−/−^* mice.

## Results

### Hypoxia-Ischemia Increases GFAP mRNA Expression

**Figure 1:**
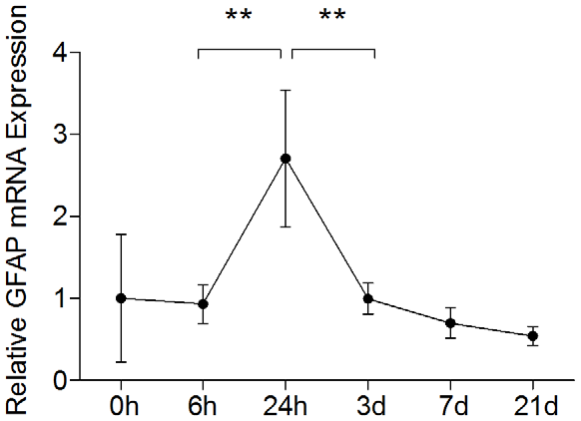
GFAP mRNA expression in mice after hypoxia-ischemia, assessed by quantitative rt-PCR. Cortex from wild-type mice exposed to hypoxia-ischemia on P9, examined by quantitative rt-PCR immediately after (n = 6), 6 h (n = 4), 24 h (n = 3), 3d (n = 4), 7d (n = 4), and 21d (n = 4) after hypoxia-ischemia. The values represent a fold increase compared to 0 hours after HI. Data are mean ± SD (** p<0.01).

To assess the effect of hypoxia-ischemia (HI) on GFAP expression in wild-type mice, we measured relative GFAP mRNA expression immediately after, 6 hours, 24 hours, 3 days, 7 days and 21 days after HI. Twenty four hours after HI, GFAP mRNA expression in the cortex was substantially increased (2.7±0.8, n = 3) compared to 6 hours (0.9±0.2, n = 4, p<0.01) or 3 days after HI (1.0±0.2, n = 4, p<0.01; Fig. 1). These data show that, astrocytes respond to HI by up-regulation of GFAP, within the first 24 hours after ischemic insult.

### The Absence of GFAP and Vimentin Does Not Affect Brain Growth or Infarct Volume after Hypoxia-Ischemia

**Figure 2:**
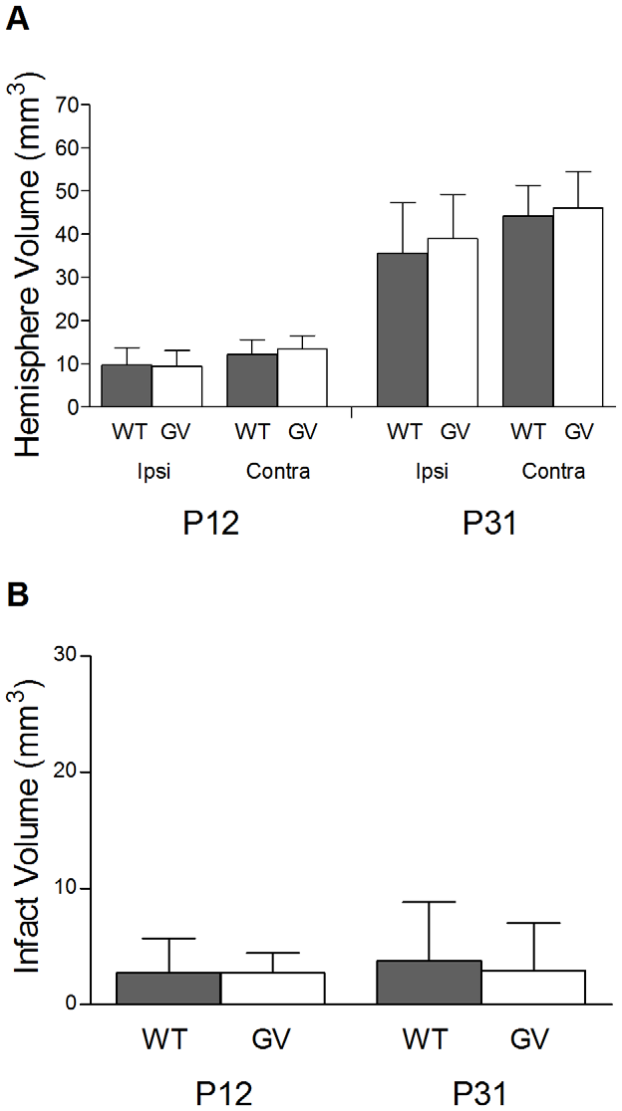
Hemisphere and infarct volume in *GFAP^−/−^Vim^−/−^* and wild-type mice after hypoxia-ischemia. Volume measurements in MAP-2 stained sections from *GFAP^−/−^Vim^−/−^* (GV) (n = 23) and wild-type (WT) (n = 19) mice at P12 and P31, i.e. 3 and 22 days after hypoxia-ischemia. (A) Ipsi- and contralateral hemisphere volume in *GFAP^−/−^Vim^−/−^* and wild-type mice. (B) Infarction volume in *GFAP^−/−^Vim^−/−^* and wild-type mice. Data are mean ± SD.

**Figure 3:**
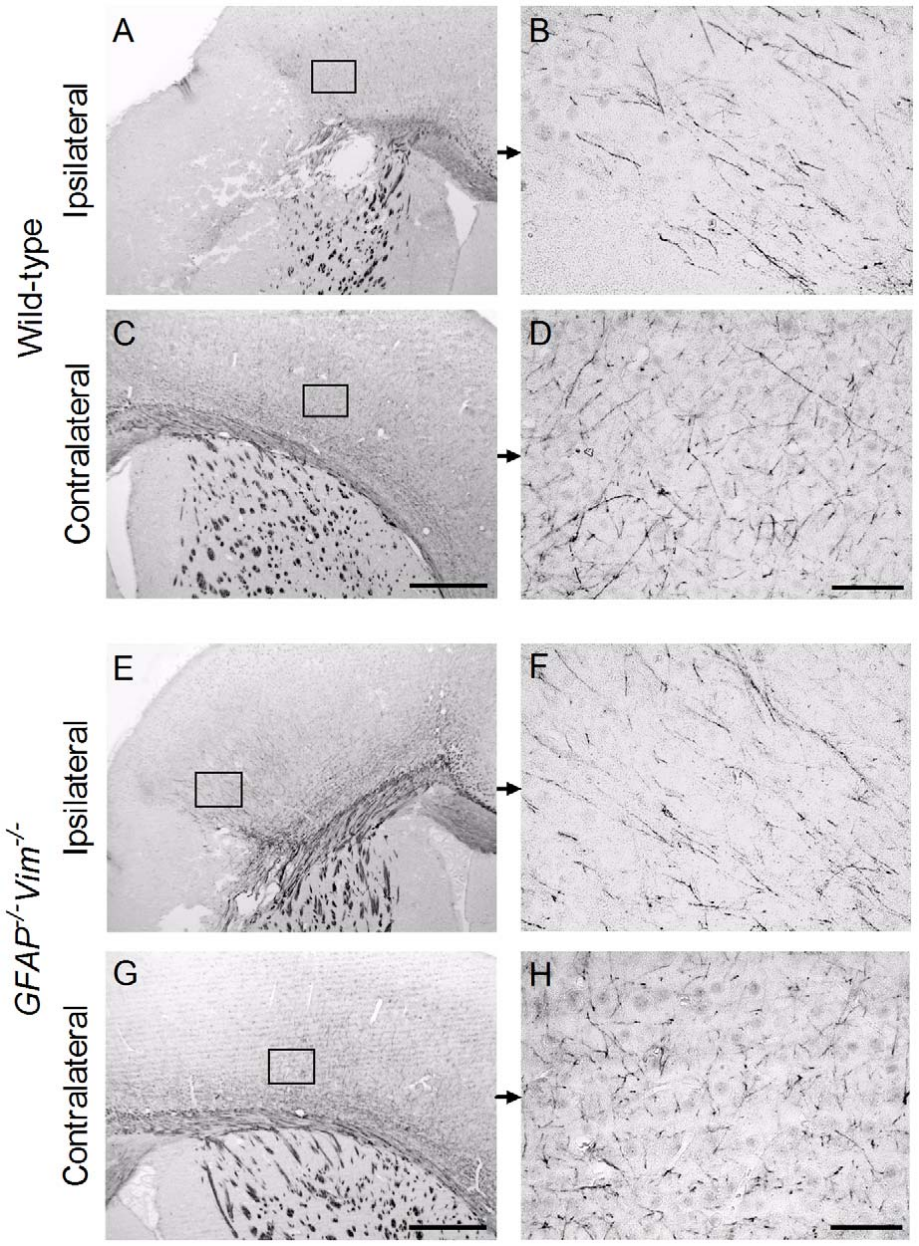
Visualization of neuronal axons in the infact area and in the contralateral hemisphere. Neuronal axons were visualized with antibodies against neurofilament proteins on sections from wild-type (A-D) and *GFAP^−/−^Vim^−/−^* (E-H) mice at P12, i.e. 3 days after hypoxia-ischemia. The frames indicate respective fields for higher magnification. Scale bar for A, C, E, G = 500 µm; for B, D, F, H = 50 µm.

Next, we assessed the hemisphere and infarct volume in *GFAP^−/−^Vim^−/−^* and wild-type mice at 3 and 22 days after ischemia. Three days after HI (P12), there was no difference in the volume of the ipsilateral hemisphere between *GFAP^−/−^Vim^−/−^* and wild-type mice (9.4±3.7 mm^3^, n = 23 *vs*. 9.7±4.1 mm^3^, n = 19). The volume of the contralateral hemisphere was comparable in *GFAP^−/−^Vim^−/−^* and wild-type mice (13.5±3.1 mm^3^ *vs*. 12.2±3.5 mm^3^) (Fig. 2a). Further, there was no difference in the volume of the ipsilateral hemisphere (38.9±10.3 mm^3^, n = 26 *vs*. 35.5±11.7 mm^3^, n = 27) or the contralateral hemisphere (46.0±8.5 mm^3^ *vs*. 44.2±7.1 mm^3^) between *GFAP^−/−^Vim^−/−^* and wild-type mice at P31, i.e. 22 days after HI (Fig. 2a). We did not find any difference in the infarct volume between *GFAP^−/−^Vim^−/−^* and wild-type mice at P12 (2.7±1.7 mm^3^, n = 23 *vs*. 2.7±3.0 mm^3^, n = 19) or at P31 (2.9±4.1 mm^3^, n = 26 *vs*. 3.7±5.1 mm^3^, n = 27; Fig. 2b). Next, we used immunohistochemistry with antibodies against neurofilament proteins to visualize neuronal axons in the vicinity of the infact and in the corresponding region in the contralateral hemisphere. We did not find any difference in the apperence of neuronal axons between the *GFAP^−/−^Vim^−/−^* and wild-type mice (Fig. 3).

### The GFAP^−/−^Vim^−/−^ Mice Have Higher Cortical Neurogenesis after Hypoxia-Ischemia

**Figure 4:**
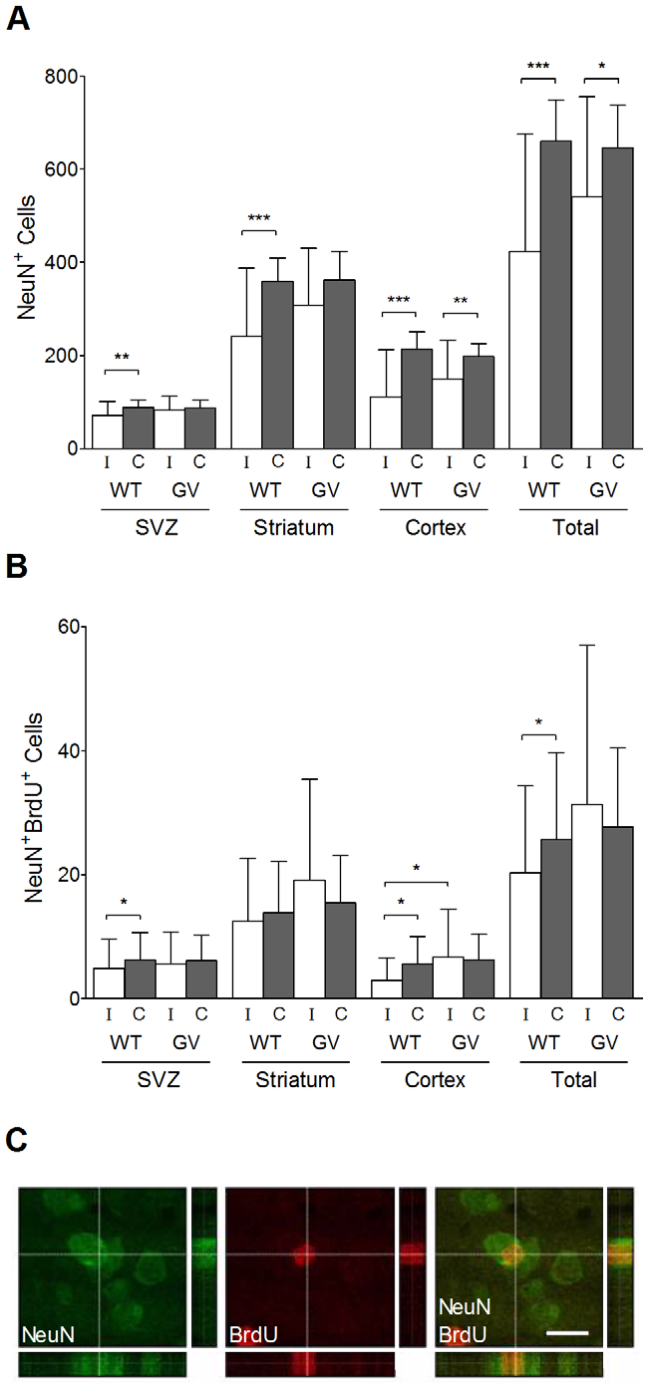
Quantification of NeuN^+^ and NeuN^+^BrdU^+^ cells at 22 days after hypoxia-ischemia. (A) Number of NeuN^+^ cells per optical field in the ipsi- and contralateral subventricular zone, striatum and cortex, in *GFAP^−/−^Vim^−/−^* (GV) (n = 29) and wild-type (WT) (n = 28) mice at P31, i.e. 22 days after hypoxia-ischemia. (B) Number of NeuN^+^BrdU^+^ cells per optical field in ipsi- and contralateral subventricular zone, striatum and cortex, in *GFAP^−/−^Vim^−/−^* (n = 29) and wild-type (n = 28) mice at P31, i.e. 22 days after hypoxia-ischemia. The numbers represent cells counted on 8 µm thick stacks of confocal images within the 450 µm×300 µm optical fields in the respective brain regions. (C) Orthogonal sections showing NeuN^+^BrdU^+^ cells. Data are mean ± SD. (* p<0.05, ** p<0.01, *** p<0.001). Scale bar = 15 µm.

To assess the effect of HI on neuronal survival outside the infarct, we counted the NeuN^+^ neurons in the subventricular zone region, striatum and cortex. As a measure of HI-induced neurogenesis, we counted NeuN^+^BrdU^+^ cells (surviving newly born neurons) in the same brain regions. We did not find any difference in the number of NeuN^+^ neurons in the ipsilateral (539.6±215.7, n = 29 *vs*. 423.6±251.9 per optical field, n = 28) or contralateral (646.0±90.5 *vs*. 660.2±87.9 per optical field) hemisphere between *GFAP^−/−^Vim^−/−^* and wild-type mice at P31 (Fig. 4a). In wild-type mice, the number of NeuN^+^ neurons in the contralateral hemisphere compared to the ipsilateral hemisphere was higher in the subventricular zone (88.3±16.3 *vs*. 70.4±31.0 per optical field, p<0.01), striatum (358.8±50.7 *vs*. 241.4±146.0 per optical field, p<0.001) and cortex (213.1±37.6 *vs*. 111.8±99.9 per optical field, p<0.001). In *GFAP^−/−^Vim^−/−^* mice, a higher number of NeuN^+^ neurons in contralateral compared to the ipsilateral hemisphere was observed in cortex (197.7±28.0 *vs*. 149.5±82.9 per optical field, p<0.01; Fig. 4a) but not in the other regions. The *GFAP^−/−^Vim^−/−^* mice had an increased number of NeuN^+^BrdU^+^ cells in ipsilateral cortex compared to wild-type mice (6.7±7.7, n = 29 *vs*. 2.9±3.6 per optical field, n = 28, p<0.05). Compared to the ipsilateral hemisphere, the wild-type mice had a higher number of NeuN^+^BrdU^+^ neurons in the contralateral subventricular zone (4.9±4.7 *vs*. 6.3±4.4 per optical field, p<0.05) and cortex (2.9±3.6 *vs*. 5.6±4.4 per optical field, p<0.05; Fig. 4b). *GFAP^−/−^Vim^−/−^* and wild-type mice not exposed to HI had a very small number of NeuN^+^BrdU^+^ neurons in the subventricular zone (0.5±0.4, n = 12 *vs*. 0.5±0.6 per optical field, n = 6), striatum (0.6±0.6 *vs* 0.1±0.2 per optical field) and cortex (0 *vs*. 0.1±0.2 per optical field).

### Hypoxia-Ischemia Leads to the Loss of Astrocytes in Wild-Type but Not GFAP^−/−^Vim^−/−^ Mice

**Figure 5:**
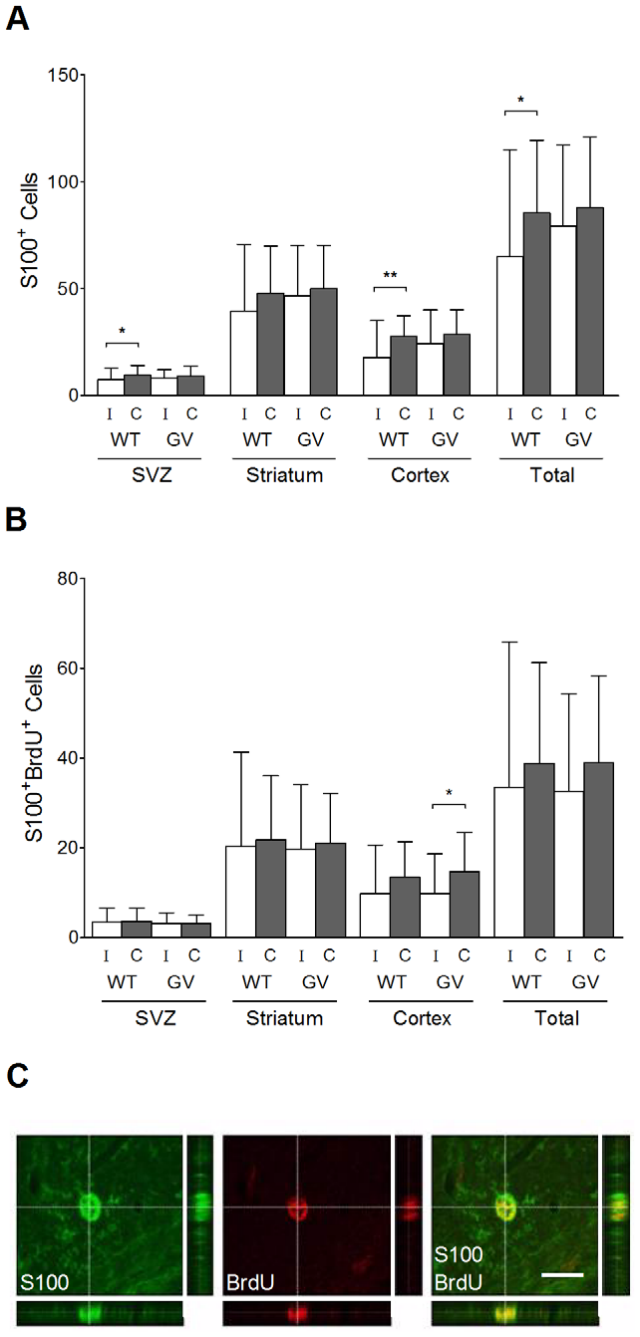
Quantification of S100^+^ and S100^+^BrdU^+^ cells at 22 days after hypoxia-ischemia. (A) Number of S100^+^ cells per optical field in ipsi- and contralateral subventricular zone, striatum and cortex, in *GFAP^−/−^Vim^−/−^* (GV) (n = 29) and wild-type (WT) (n = 28) mice at P31, i.e. 22 days after hypoxia-ischemia. (B) Number of S100^+^BrdU^+^ cells per optical field in ipsi and contralateral subventricular zone, striatum and cortex, in *GFAP^−/−^Vim^−/−^* (n = 29) and wild-type (n = 28) mice at P31, i.e. 22 days after hypoxia-ischemia. The numbers represent cells counted on 8 µm thick stacks of confocal images within the 450 µm×300 µm optical fields in the respective brain regions. (C) Orthogonal sections showing S100^+^BrdU^+^ cells. Data are mean ± SD. (* p<0.05, ** p<0.01). Scale bar  = 15 µm.

Next, we assessed the effect of HI on astrocyte numbers and counted S100^+^ astrocytes in the subventricular zone region, striatum and cortex. As a measure of HI-induced astrogenesis, we counted S100^+^BrdU^+^ cells (surviving newly born astrocytes) in the same brain regions. In *GFAP^−/−^Vim^−/−^* mice, the number of S100^+^ astrocytes did not differ between the ischemic and contralateral hemisphere at P31 (Fig. 5a). In wild-type mice, the number of S100^+^ astrocytes was lower in the ipsilateral compared to contralateral hemisphere (65.0±50.1 *vs*. 85.6±34.0 per optical field, p<0.05), both in subventricular zone (7.5±5.4 *vs*. 9.7±4.7 per optical field, p<0.05) and in the cortex (18.0±17.1 vs. 27.8±9.5 per optical field, p<0.01; Fig. 5a). The *GFAP^−/−^Vim^−/−^* and wild-type mice did not differ in the histomorphology of S100^+^ astrocytes as assessed by the length of the longest cellular process of astrocytes in the cortex around the infarct area at P31 (16.0±1.5 µm, n = 6 vs. 16.5±0.6 µm, n = 6). The *GFAP^−/−^Vim^−/−^* and wild-type mice did not differ in number of S100^+^BrdU^+^ cells (surviving newly born astrocytes) (Fig. 5b).

## Discussion

Astrocytes respond to hypoxia-ischemia (HI) by morphological and functional changes as early as 8 hours after the insult and have been implicated to play a role in the pathophysiological events that lead to the brain damage after HI [15], [16]. To investigate the role of reactive astrocytes in response to hypoxic-ischemic brain injury in the immature brain, we used mice in which astrocyte activation is attenuated due to the lack of intermediate filament proteins GFAP and vimentin [3], [8] and subjected them to neonatal HI. We found that attenuation of reactive gliosis does not have any effect on the infarct volume or the volume of the injured hemisphere, neither at an early (3 days) nor a late (22 days) time point. Thus, in contrast to the adult brain, where reactive astrocytes play a critical role in protecting the ischemic penumbra [17], in the immature brain reactive gliosis does not seem to play a major neuroprotective role. The immature and adult brain differ in their responses to ischemic injury, such as leukocyte transmigration [18], activation of microglia [19], cytokine and chemokine interplay [20], excitotoxicity [21] and mitochondria-regulated apoptotic mechanisms [22], [23]. The present results support the notion that also the role of astrocytes in the pathogenesis of ischemic brain injury depends on the developmental age which has to be taken into consideration in designing future neuroprotective strategies.

We have also found that both the wild-type and the *GFAP^−/−^Vim^−/−^* mice responded to HI by increased production of new neurons. Interestingly, the wild-type mice showed a lower number of surviving newly born neurons in the ischemic subventricular zone and the cortex compared to the contralateral hemisphere, whereas in the *GFAP^−/−^Vim^−/−^* mice the neurogenic response to ischemia was the same in the two hemispheres. Since neuronal cell loss in the ischemic hemisphere was comparable between the wild-type and *GFAP^−/−^Vim^−/−^* mice, it is conceivable that the absence of reactive gliosis results in a niche that is more permissive to neurogenesis and the survival of newly formed neurons after ischemia. Indeed, we have earlier shown that survival and integration of neuronal grafts is increased in the CNS of *GFAP^−/−^Vim^−/−^* mice [24], [25] and that basal hippocampal neurogenesis and cell proliferation is increased in aged *GFAP^−/−^Vim^−/−^* mice [26].

Remarkably, we found that HI led to the loss of S100^+^ astrocytes in the ischemic hemisphere of wild-type but not *GFAP^−/−^Vim^−/−^* mice. The *GFAP^−/−^Vim^−/−^* and wild-type mice did not differ in the number of surviving newly formed astrocytes. These findings suggest that the astrocytes lacking GFAP and vimentin might be less vulnerable to ischemic stress. Indeed, the dynamics of the response of *GFAP^−/−^Vim^−/−^* astrocytes to hypotonic stress, a component of HI, might be altered. It was previously shown that *GFAP^−/−^Vim^−/−^* astrocytes in vitro reacted to hypotonic stress by releasing less taurine than wild type astrocytes [27] and that *GFAP^−/−^Vim^−/−^* astrocytes exhibited more prolonged changes in extracellular space diffusion parameters than wild-type astrocytes during cell swelling evoked by hypotonic stress or high concentration of potassium [28].

In summary, our data suggest that in contrast to its protective role in adult focal brain ischemia, reactive gliosis does not seem to play a major role in the protection of brain tissue against HI injury in the immature brain. Thus, the role of reactive astrocytes in the pathogenesis of ischemic brain injury might be profoundly affected by age at the time of insult.

## Materials and Methods

### Ethics Statement

The animal experiments were approved by the local Animal Ethics Committee at the University of Gothenburg (Ethical approval 29-2006).

### Animals and Surgery

The *GFAP^−/−^Vim^−/−^* and wild-type mice were on a mixed C57BL6/129SV/129Ola genetic background [8], [10], [29]. Neonatal HI was induced on P9, as previously described with modifications for mice [12], [13], [14]. The mice were anesthetized with isoflurane (Baxter Medical), 3.5% for the induction and 1.5% to continue, in 50% oxygen and 50% nitrous oxide. The left common carotid artery was dissected and permanently ligated with a prolene suture (6.0). The incision was closed and infiltrated with lidokain (Xylocain®, Astra Zeneca). The mice were injected intraperitoneally (i.p.) with bromodeoxyuridine (BrdU) (Sigma-Aldrich) (25 mg/kg body weight), dissolved in 0.9% NaCl saline twice daily from P9 to P12. The mice were then returned to their mothers for 1 hour and then placed in a chamber with humidified air at 36°C for 10 minutes and exposed to humidified 10% oxygen in nitrogen, for 30 minutes at 36°C and kept in humidified air at 36°C for 10 additional minutes before returning to their mothers.

### Immunohistochemistry

The mice were deeply anesthetized with thiopenthal (50 mg/ml) (Pentothal® Sodium, Hospira) i.p. on P12 or P31. The mice were perfusion fixed intracardially with 0.9% NaCl followed by 5% paraformaldehyde (Histofix, Histolab). The brains were removed and post-fixed in 5% paraformaldehyde for 24 hours at 4°C, placed in 70% ethanol for 24 hours at room temperature (RT), dehydrated and embedded in paraffin. The brains were cut in 8 µm coronal sections using a water microtome (Rotary Microm HM 355S, Microm International). For immunohistochemical staining, sections were deparaffinised in xylene and alcohol and boiled in 0.01 M citric acid buffer (pH 6.0) for 10 minutes, incubated in phosphate buffered saline (PBS) with 3% H_2_O_2_ for 10 minutes, in 2 M HCl for 1 hour and then 30 minutes in PBS with 0.2% Triton X-100 and 3% BSA.

For brain infarction area evaluation, the primary monoclonal mouse anti-microtubule-associated protein-2 (MAP-2) antibody (Sigma-Aldrich) 1∶2000 and anti-pan-neurofilament protein (SMI 312) antibody (Covance) 1∶2000 were used with the secondary horse-anti-mouse, BA-2001 antibody (Vector Laboratories) 1∶250. Sections were exposed to avidin-biotin enzyme complex ABC-Elite (Vector Laboratories) for 1 hour and visualized with 3,3-diaminobenzidine (DAB; 0.5 mg/ml) and nickel sulphate (15 mg/ml).

The following antibodies were used for fluorescent staining at the following dilutions: primary monoclonal mouse anti-BrdU antibody (Dako) 1∶100 with the secondary goat anti-mouse Alexa Fluor 594 antibody (Invitrogen) 1∶500, primary monoclonal mouse anti-NeuN biotin conjugated antibody (Chemicon) 1∶100 with the secondary Streptavidin Alexa Fluor 488 antibody (Invitrogen) 1∶500, primary polyclonal rabbit anti-S100 antibody (Dako) 1∶200 with the secondary goat anti-rabbit Alexa Fluor 488 antibody (Invitrogen) 1∶500. The primary antibody solution was left on the sections over night, in a dark room at 4°C. The sections were washed in PBS and the secondary antibody solution was added for 1 hour at RT. Finally, the sections were washed in PBS and mounted with Dako fluorescent mounting medium.

### Brain Infarction Area Evaluation

A CCD camera (Olympus DP50) connected to a microscope (Optiphot-2, Nikon) was used to obtain images of the MAP-2 stained brain sections for calculation of the infarct volume and hemisphere volume. Olympus Micro Image, v4.0 software was used to delineate MAP-2 positive and negative areas of contralateral and ipsilateral hemisphere, on sections with 320 µm interval, throughout the brain. The infarct volume was assessed as the MAP-2 negative area in the ipsilateral hemisphere and the ipsilateral hemisphere volume was assessed as the MAP-2 negative area plus the ipsilateral MAP-2 positive area.

### Cell Counting and Astrocyte Histomorphology

Images of brain sections double stained with fluorescent antibodies were captured through 3-dimensional Z-series consecutive scans with fluorescent signals detected and processed by a confocal scanning laser microscope (Leica TCS SP2, Leica Microsystems). Three images, representing three horizontal brain regions, were sampled at the level of the striatum, starting medially from the subventricular zone (SVZ), through the dorsal striatum and laterally to the cortex. ImageJ (Image Processing and Analysis in Java) 1.40 software was used to analyse the Z-series stacks. Fluorescent positive cells were counted on 8 µm thick stacks of confocal images within the 450 µm×300 µm optical fields in the respective brain regions. The length of the longest cellular processes of cortical astrocytes around the infarct area was measured using a Leica DM 6000B microscope with a 40× objective and Stereoinvestigator 9 (MicroBrightField System, Inc) on 8 µm thick S100 immunostained sections, as previously described [11].

### Quantitative real-time PCR

Wild-type mice were killed at 0 hours, 6 hours, 24 hours, 3 days, 7 days and 21 days after hypoxia-ischemia. The hippocampus, striatum and cortex were dissected out and were quickly frozen on dry-ice and store at -80°C. Total RNA was extracted using RNeasy Lipid Tissue Mini Kit, including DNase treatment (Qiagen). RNA concentrations were measured with a NanoDrop ND-1000 spectrophotometer (Nanodrop Technologies) and RNA integrity was checked on some randomly picked samples using the Agilent 2100 bioanalyzer (Agilent Technologies). Reverse transcription was performed with SuperScript III (Invitrogen) according to the instructions of the manufacturer using a mixture of 2.5 µM oligo (dT) and 2.5 µM random hexamers (both Invitrogen) as primers. Temperature profile for reverse transcription was 25°C for 5 minutes, 50°C for 60 minutes, 55°C for 15 minutes, and 70°C for 15 minutes. Real-time PCR measurements were performed using a LightCycler 480 (Roche Diagnostics). Temperature profile of real-time PCR was 95°C for 3 minutes, followed by 50 cycles at 95°C for 20 seconds, 60°C for 20 seconds, and 72°C for 20 seconds. Ten-microliter reactions contained iQ SYBR Green Supermix (Bio-Rad) and 400 nM of each primer (Eurofins MWG Operon). The following primer sequences were used: *Gfap*_fwd: AACCGCATCACCATTCCT and *Gfap*_rev: CGCATCTCCACAGTCTTTACC. Reference genes were evaluated using the Mouse Endogenous Control Gene Panel (TATAA Biocenter) and NormFinder [30]. All data were geometrically averaged against *Pgk1* and *B2m*. Formation of correctly sized PCR products was confirmed by agarose gel electrophoresis (2%) for all assays and melting curve analysis for all samples. Data analysis was performed as previously described [31], [32].

### Statistics

The Student t-test and Mann Whitney test was used for statistical analysis between different time points or between the animal groups (GraphPad Prism). The Wilcoxon signed rank test was used to compare data between brain hemispheres from the same animal. Results are given as mean ± standard deviation (SD). Statistical differences were considered significant if p*<*0.05.
